# Interplay Between Angiotensin II Type 1 Receptor and Thrombin Receptor Revealed by Bioluminescence Resonance Energy Transfer Assay

**DOI:** 10.3389/fphar.2020.01283

**Published:** 2020-08-20

**Authors:** Isra Al Zamel, Abdulrasheed Palakkott, Arshida Ashraf, Rabah Iratni, Mohammed Akli Ayoub

**Affiliations:** Department of Biology, College of Science, The United Arab Emirates University (UAEU), Al Ain, United Arab Emirates

**Keywords:** angiotensin II, AngII type 1 receptor, thrombin, protease-activated receptor 1, G protein-coupled receptors, bioluminescence resonance energy transfer, vascular system, heterodimerization

## Abstract

The key hormone of the renin-angiotensin system (RAS), angiotensin II (AngII), and thrombin are known to play major roles in the vascular system and its related disorders. Previous studies reported connections between AngII and thrombin in both physiological and pathophysiological models. However, the molecular mechanisms controlling such interplay at the level of their receptors belonging to the family of G protein-coupled receptors (GPCRs) are not fully understood. In this study, we investigated the functional interaction between the AngII type 1 receptor (AT1R) and the thrombin receptor [or protease-activated receptor 1 (PAR1)] in human embryonic kidney 293 (HEK293) cells. For this, we used various bioluminescence resonance energy transfer (BRET) proximity-based assays to profile the coupling to the heterotrimeric Gαq protein, β-arrestin recruitment, and receptor internalization and trafficking in intact cells. The overall dose-response and real-time kinetic BRET data demonstrated the specific molecular proximity between AT1R and PAR1 resulting in their functional interaction. This was characterized by thrombin inducing BRET increase within AT1R/Gαq and AT1R/β-arrestin pairs and synergistic effects observed upon the concomitant activation of both receptors suggesting a positive allosteric interaction. The BRET data corroborated with the data on the downstream Gαq/inositol phosphate pathway. Moreover, the selective pharmacological blockade of the receptors revealed the implication of both AT1R and PAR1 protomers in such a synergistic interaction and the possible transactivation of AT1R by PAR1. Interestingly, the positive action of PAR1 on AT1R activation was contrasted with its apparent inhibition of AT1R internalization and its endosomal trafficking. Finally, BRET saturation and co-immunoprecipitation assays supported the physical AT1-PAR1 interaction in HEK293 cells. Our study reveals for the first time the functional interaction between AT1R and PAR1 *in vitro* characterized by a transactivation and positive allosteric modulation of AT1R and inhibition of its desensitization and internalization. This finding may constitute the molecular basis of the well-known interplay between RAS and thrombin. Thus, our data should lead to revising some findings on the implication of RAS and thrombin in vascular physiology and pathophysiology revealing the importance to consider the functional and pharmacological interaction between AT1R and thrombin receptors.

## Introduction

In physiology, the interplay between different signal molecules such as hormones, neurotransmitters, and cytokines occurs at the level of their respective receptors in the different cells and tissues (Barnes, 2006). This has been extensively documented for the family of G protein-coupled receptors (GPCRs) for which the functional and/or the physical interaction, more often referred to heterodimerization, has been reported *in vitro* and *in vivo* (Prinster et al., 2005; Gomes et al., 2016). At the molecular and cellular levels, such a phenomenon is characterized by either a direct or indirect interaction between the different receptors, a crosstalk, or trans-modulation between them resulting in changes of receptor pharmacology and signaling with an implication on their specific cellular and physiological responses (Prinster et al., 2005; Prezeau et al., 2010; Gomes et al., 2016). Furthermore, the interaction between GPCRs is intertwined with the concept of allostery and allosteric modulation between hormones and their receptors (Springael et al., 2006; Haack and McCarty, 2011). Consequently, GPCR interplay and interaction have evolved rapidly over the recent years, bringing more evidence for the importance of the functional/physical interaction between GPCRs in physiology and pathophysiology and thus impacting our view on drug discovery and their targeting in human diseases (Prinster et al., 2005; Dalrymple et al., 2008).

One of the interesting interplays that has been reported in many *in vitro* and *in vivo* studies is the one involving AngII and the protease thrombin (Weber, 1994; Polevaya, 1996; Kim and Iwao, 2011; Hasan et al., 2019). AngII and thrombin are two key regulators in the vascular system by regulating blood flow and pressure and the coagulation, respectively, with implications in many vascular disorders including hypertension, atherosclerosis, and restenosis after balloon angioplasty (Viswanathan et al., 1992; Ross, 1993; Wilcox et al., 1994; Ishida et al., 1999). At the cellular levels, both AngII and thrombin are known to activate diverse signaling pathways controlling the proliferation, hypertrophy, and the migration of vascular cells (McNamara et al., 1996; Capers et al., 1997; Griendling et al., 1997).

The AngII/thrombin interplay has been evidenced in different physiological (platelet aggregation, vasoconstriction, salt and water reabsorption, proliferation of vascular muscle cells, and chemotaxis) and pathophysiological (atherosclerosis, restenosis, hypertension, and glomerular diseases) models (Weber, 1994; Polevaya, 1996; Kim and Iwao, 2011; Hasan et al., 2019). Early studies have suggested the implication of both AngII and thrombin in platelet aggregation, chemotactic effects, vasoconstriction, increased permeability of the arterial vessels, sodium and water retention, proliferation of the vascular smooth muscle cells (VSMC) and fibroblasts (Whitaker et al., 1973; Weber, 1994). Other evidence indicated that thrombin has two opposing vascular effects: endothelium-dependent relaxation and endothelium-independent vasoconstriction (Walz et al., 1985; Ku and Zaleski, 1993; Polevaya, 1996). Besides, smooth muscle cell proliferation in response to AngII and thrombin is involved in the pathology of post-percutaneous trans-luminal coronary angioplasty (PTCA) restenosis in animal models (Pawlowski et al., 1997). Recently, the thrombin-mediated atrial endothelial senescence was reported to be associated with an overexpression of angiotensin-converting enzyme (ACE) and AngII receptor (AT1R) (Hasan et al., 2019). Hence, targeting thrombin and angiotensin systems has been proposed as an approach to prevent atrial endothelial senescence. At the molecular level, one *in vivo* study using the hypertensive rat model showed an increase of vascular thrombin receptor mRNA mediated by AngII (Capers et al., 1997). Such an increase was observed especially in the AT1R-rich vascular smooth muscle layer of rats suggesting the implication of both AngII and thrombin in atherosclerosis (Capers et al., 1997). Similarly, AngII increased the expression of thrombin receptors in cultured and native VSMC speculating on the possible synergistic effect between AT1R and thrombin receptors to potentiate the mitogenic activity of thrombin and the proliferation of VSMC during vascular injury (Fisslthaler et al., 1998). Moreover, both thrombin and AngII have been shown to activate the mitogenic pathways in NIH3T3 cells *via* differential phosphorylation pattern of Raf-1 and Shc (Apostolidis and Weiss, 1997). In VSMC, AngII and thrombin, respectively *via* AT1R and PAR1, can also induce the expression of vascular endothelial growth factor (VEGF) and its receptors promoting angiogenic responses (Williams et al., 1995; Richard et al., 2000; Richard et al., 2001). Recently, thrombin-mediated atrial endothelial senescence is associated with an overexpression of AT1R and this was inhibited by AT1R blocker (Hasan et al., 2019). Together, these observations on the interplay between AngII and thrombin illustrate the importance of considering the functional interaction between these two factors at the level of their specific receptors. All these studies were interpreted in the context where the putative interaction between AngII and thrombin at the level of their receptors was not fully considered. More importantly, the therapeutic strategies involving RAS are mostly based on AngII receptor blockers combined with ACE inhibitors (Heyndrickx, 1993; Serruys et al., 1995; Pratt Richard and Dzau Victor, 1996; Tsukuda et al., 2011; Urushihara et al., 2011). Therefore, the objective of the present study was to investigate the putative functional interaction between AT1R and thrombin and its receptor, PAR1, *in vitro* by assessing the pharmacological and functional effects of thrombin and PAR1 on AT1R activation and signaling, internalization, and endosomal trafficking, as well as their physical interaction, in HEK293 cells using various BRET proximity-based assays as previously described (Ayoub, 2016).

## Materials and Methods

### Chemical and Reagents

The following human protein-coding plasmids were used for the transient expression in HEK293 cells: PAR1was purchased from cDNA Resource Center (Bloomsberg, PA, USA), PAR1-Rluc was used as previously described (Ayoub et al., 2010), HA-AT1R was kindly provided by Reiter E. (INRA, Nouzilly, France), AT1R-Rluc was a gift from Laporte, S. (McGill University, Montréal, Canada), yPET-β-arrestin 2 was kindly provided by Scott, M. (Cochin Institute, Paris, France), Grb2-Rluc and Grb2-Venus were kindly given by Prof. Pfleger KD. (Harry Perkins Institute of Medical Research and UWA, Perth, Australia), and Venus-Gαq, Venus-Kras, and Venus-Rabs that were generously shared by Lambert, N. (Augusta University, GA, USA). AngII, thrombin, and irbesartan were from Sigma (St Louis, MO, USA). SCH79797 was from Tocris (Tocris, Ellisville, MO). The IP1 kit was purchased from Cisbio Bioassays (PerkinElmer, Codelet, France) and the anti-HA tag antibody (ab9110) was purchased from abcam (abcam, MA, USA).

### Cell Culture and Transfection

HEK293 cells were maintained at 37°C, 5% CO2 in complete medium (Dulbecco’s modified Eagle’s medium (DMEM) containing 0.3 mg/ml glutamine, 100 IU/ml penicillin, and 100 µg/ml streptomycin) supplemented with 10% fetal calf serum (GIBCO BRL, Carlsbad, CA). Transient transfections were carried out using Lipofectamine™ 2000 (Invitrogen) as previously described (Ayoub et al., 2010; Ayoub et al., 2015a; Ayoub et al., 2015b). For BRET and IP1 assays, cells were directly transfected in 96-well white plates. Briefly, for each 96-well 25 ng of AT1R-Rluc coding plasmid was mixed with 25 ng of Venus-Gαq, yPET-β-arrestin 2, Venus-Kras, or Venus-Rab5/7 coding plasmid without or with 25 ng of untagged PAR1 coding plasmid in 25 μl of serum-free DMEM. In parallel, 0.5 µl per well of Lipofectamine™ 2000 was mixed in 25 µl of serum-free DMEM. After incubation for 5 min at room temperature, the plasmid solution was mixed with Lipofectamine solution and incubated for 20 min at room temperature. Then, the transfection mix (50 µl) was directly added to the cells (5 x 10^4^ cells per well) in a final volume of 200 µl of culture medium and seeded in 96-well white plate. For co-immunoprecipitation, 7.5 x 10^6^ cells were cultured in T-75 flasks and transfected with the total of 10 μg of plasmids (5 μg of HA-AT1R and 5 μg of AT1R-Rluc, PAR1-Rluc, or Grb2-Rluc) resuspended in 500 µl of serum-free DMEM using 5 µl of Lipofectamine previously mixed with 500 µl of serum-free DMEM. All assays were carried out 48 h posttransfection.

### Dose-Response BRET Assay

BRET technology was used as previously described (Ayoub et al., 2010; Ayoub et al., 2015a; Ayoub et al., 2015b) using 40 μl as the total reaction volume per well. Cells co-expressing the different BRET donors and acceptors were first seeded in 96-well white plates, washed with 50 μl/well of PBS, and treated for 30 min at 37°C with 40 μl of PBS containing or not the indicated doses of either AngII, thrombin, or both, as indicated. Following treatment, 10 μl of coelenterazine h (Promega Corporation, Madison, WI, USA) was added to a final concentration of 2.5 µM and BRET measurements were carried out using the Tristar 2 multilabel plate reader (Berthold, Germany) that allows the sequential integration of light emission detected with two filter settings (480 ± 20 nm and 540 ± 25 nm).

### Real-Time BRET Kinetics

Cells co-expressing the different BRET donors and acceptors were first seeded in 96-well white plates and washed with 50 μl/well of PBS. Then, cells were resuspended in 30 μl of PBS followed by addition 10 μl of coelenterazine h (2.5 µM), and BRET signals were measured for 5 min before and 55 min after the addition of 10 μl of either AngII, thrombin, or both, as indicated.

### BRET Assay With Antagonists

Cells co-expressing the different BRET donors and acceptors were first seeded in 96-well white plates, washed with 50 μl/well of PBS, and resuspended in 30 μl of PBS containing or not (control) SCH-79797 (100 μM) or irbesartan (10 μM) and incubated for 15 min at 37°C. Then, 10 μl of either AngII, thrombin, or both were added followed by further incubation for 30 min at 37°C and addition 10 μl of coelenterazine h (2.5 µM) before BRET signals were measured.

### BRET Saturation Assay

Cells co-expressing a constant amount of AT1R-Rluc (25 ng) without or with increasing amount of either PAR1-Venus or Grb2-Venus (10, 20, 40, 60, 80, 100, 120, 140, 160, 175 ng) were first seeded in 96-well white (for BRET and luminescence measurements) and black (for fluorescence measurements) plates, washed with 50 μl/well of PBS, and resuspended in 40 μl of PBS. Then, 10 μl of coelenterazine h (2.5 µM) were added and BRET signals were measured. In parallel, the specific luminescence of AT1R-Rluc was measured using a 485 nm emission filter, and the specific fluorescence of PAR1-Venus and Grb2-Venus was quantified using the 485 nm excitation filter and 540 nm emission filter on the Tristar 2 multilabel plate reader. Then, net BRET signals were correlated and plotted over the fluorescence/luminescence ratios (Venus/Rluc ratios).

### IP1 Assay

Measurement of IP1 accumulation was performed using the IP-One Tb kit (Cisbio Bioassays, France) according to the manufacturer’s instructions. For this, cells were first pre-treated or not (Control) with 30 µl of SCH-79797 (100 μM) or irbesartan (10 μM) in the stimulation buffer 1X and incubated for 15 min at 37°C. Then, 10 μl of either AngII (10 nM), thrombin (1 U/ml), or both were added followed by further incubation for 30 min at 37°C. For the dose-response experiments, 40 µl of increasing doses of AngII or thrombin in the stimulation buffer 1X were used. The cells were then lysed by adding the supplied assay reagents, and the assay was incubated for 1 h at room temperature. Fluorescence emission was measured at 620 nm and 665 nm, 50 µs after excitation at 340 nm using the Tristar 2 multilabel plate reader (Berthold, Germany).

### Co-Immunoprecipitation

HEK293 cells coexpressing or not HA-AT1R with either AT1R-Rluc, PAR1-Rluc, or Grb2-Rluc were used for immunoprecipitation with the anti-HA antibody followed by the measurements of luciferase (Rluc) luminescence directly on the immunoprecipitates. The immunoprecipitation was carried out using the immunoprecipitation (Protein A) assay kit (Roche). Briefly, cells were solubilized in lysis buffer. An equal amount of each protein lysate of Rluc-tagged proteins indicated by their similar luminescence signal (2500000 a.u.) measured at 485 nm was incubated overnight with protein A agarose only for pre-clearing. Precleared lysate (1 ml) was then incubated with the anti-tag HA antibody (3 µg/ml) for 2 h at 4°C, followed by incubation with 25 µl of protein A agarose beads overnight at 4°C. Beads were washed as per the manufacturer’s instructions and the final immunoprecipitates were then resuspended in 50 µl of PBS in 96-well white plate followed by the addition of 10 μl of coelenterazine h (5 µM) as Rluc substrate. The amount of Rluc luminescence in each complex was then measured using a 485 nm emission filter on the Tristar 2 multilabel plate reader.

### Data Presentation and Statistical Analysis

The BRET data given as the ratio of light emission at 540 nm over 480 nm were first converted to “ligand-induced BRET” signals by subtracting the ratio obtained from vehicle-treated cells from the same ratio obtained from AngII/thrombin-treated cells. Then, the % of responses in the different BRET and IP1 assays were obtained by taking as 100% the maximal AngII/thrombin-induced responses in the control condition. All kinetic and the sigmoidal dose-response curves were fitted to appropriate nonlinear regression equations using GraphPad Prism software (San Diego, CA, USA). Statistical analyses were performed with two-way ANOVA and multiple comparisons test to determine statistical significance between the different conditions relative. ****p-value < 0.0001, ***p-value < 0.001, **p-value < 0.01, * p-value < 0.05, and ns p-value > 0.05.

## Results

As stated above, our study aimed to investigate the functional interaction between AT1R and thrombin and its receptor PAR1 *in vitro* by assessing the putative effects of PAR1 on the activation, the internalization, and the endosomal trafficking of AT1R in HEK293 cells. Thus, we examined the effect of thrombin treatment and the transient overexpression of PAR1 on i) the functional coupling of AT1R with its cognate heterotrimeric Gαq protein, ii) the recruitment β-arrestin 2, and iii) AT1R internalization and trafficking. For this, BRET technology using specific BRET sensors was applied as previously described (Ayoub et al., 2010; Ayoub et al., 2015a; Ayoub et al., 2015b) in live HEK293 cells coexpressing AT1R-Rluc (as BRET donor) and either Venus-Gαq, yPET-β-arrestin 2, Venus-Kras, and Venus/Rabs (as BRET acceptors) in the absence or presence of thrombin and without or with PAR1 overexpression and co-activation. Also, the Gαq/inositol phosphate pathway was investigated in both HEK293 cells transiently expressing the receptors as well as in the human epithelial colorectal adenocarcinoma cells (HT-29) reported to express endogenous AT1R (Ozeki et al., 2013; Wang et al., 2013) and PAR1 (Darmoul et al., 2004). Finally, the possible physical AT1R-PAR1 interaction was assessed by BRET saturation and co-immunoprecipitation assays using differentially-tagged receptors.

### Dose-Response Analysis Revealed the Functional Interaction Between AngII and Thrombin Through Their Receptors

**Figure 1:**
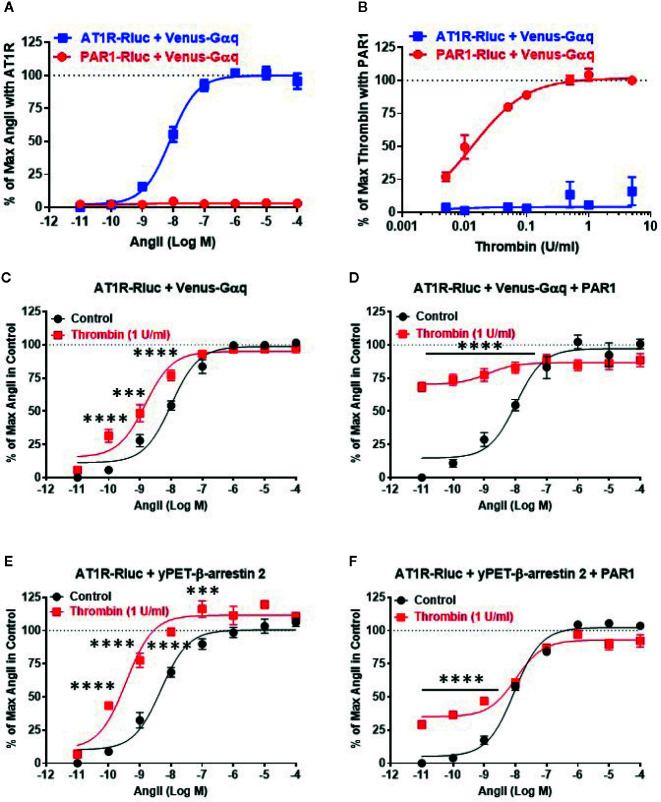
Dose-response BRET analysis of AngII-induced AT1R/Gαq coupling and AT1R/β-arrestin 2 interaction. HEK293 cells transiently coexpressing AT1R-Rluc **(A–F)** or PAR1-Rluc **(A, B)** with either Venus-Gαq **(A–D)** or yPET-β-arrestin 2 **(E, F)** in the absence or presence of PAR1 overexpression as indicated were used. Cells were then treated 30 min at 37°C with the increasing concentrations of AngII in the absence or presence of a saturating dose of thrombin (1 U/ml) before BRET signals were measured in live cells. Data are means ± SEM of three to four independent experiments performed in triplicate. The statistical analysis indicates the significance relative to the control curve (the absence of thrombin). ****p-value < 0.0001, ***p-value < 0.001.

First, we validated our BRET assays and verified the specificity of AngII and thrombin towards their respective receptors, AT1R and PAR1. Since both AT1R and PAR1 are two GPCR members known for their coupling to the heterotrimeric Gαq protein, we measured BRET signals in HEK293 cells coexpressing Venus-Gαq with either AT1R-Rluc or PAR1-Rluc and treated with increasing doses of AngII (**Figure 1A**) or thrombin (**Figure 1B**). As expected, AngII but not thrombin induced a nice dose-dependent BRET increase between AT1R-Rluc and Venus-Gαq (**Figure 1A**). Whereas, thrombin but not AngII specifically increased BRET signal between PAR1-Rluc and Venus-Gαq in a dose-dependent manner. This demonstrates the specificity of AngII and thrombin to promote BRET increase reflecting receptor-Gαq coupling with no cross-reactivity between the ligands and the receptors allowing us to make solid conclusions on the pharmacological effects of AngII and thrombin on the functional interaction between AT1R and PAR1. Also, these dose curves led to determine the EC_50_ dose of AngII (∼10 nM) and the saturating dose of thrombin (1 U/ml) to be used in the combined treatments in the different dose-response and real-time kinetic BRET and Gαq/inositol phosphate (IP1) experiments, as indicated below.

Next, we performed the dose-response analysis with AngII in the absence or presence of thrombin in cells coexpressing AT1R-Rluc and either Venus-Gαq (**Figures 1C, D**) or yPET-β-arrestin 2 without or with PAR1 overexpression as indicated. Like in **Figure 1A**, AngII promoted a nice dose-dependent BRET increase between AT1R-Rluc/Venus-Gαq (**Figures 1C, D**) and AT1R-Rluc/yPET-β-arrestin 2 (**Figures 1E, F**) pairs regardless PAR1 overexpression. In the absence of PAR1 overexpression, the stimulation of cells with the saturating dose of thrombin (1 U/ml) did not promote any significant BRET increase as shown at the baseline (**Figures 1C, E**). However and interestingly, under these conditions thrombin significantly left-shifted the dose-response curve of AngII on AT1R/Gαq (**Figure 1C**) and AT1R/β-arrestin 2 (**Figure 1E**) pairs. Indeed, for Gαq the Log EC_50_ values were -7.99 ± 0.08 and -8.82 ± 0.1 (*p-value <0.0001, n=4*) in the absence and presence of thrombin, respectively, whereas for β-arrestin 2 the Log EC_50_ values were -8.32 ± 0.10 and -9.44 ± 0.11 (*p-value <0.0001, n=3*) in the absence and presence of thrombin, respectively. This suggests a positive allosteric effect of thrombin on AT1R that may involve endogenous thrombin receptors since HEK293 cells were reported to predominantly express PAR1, PAR2, and to less extent PAR4 but only very low PAR3 (Schmidt et al., 1996; Atwood et al., 2011). To further investigate the impact of PAR1 on AT1R function, we examined the effect of PAR1 overexpression on the BRET signals reflecting AT1R/Gαq and AT1R/β-arrestin 2 coupling and interaction. The data showed that 1 U/ml of thrombin strongly promoted BRET increase (∼70%–75% of AngII response) in AT1R-Rluc/Venus-Gαq coexpressing cells (**Figure 1D**) and to less extent (∼25% of AngII response) in AT1R-Rluc/yPET-β-arrestin 2 coexpressing cells (**Figure 1F**). Interestingly, such a positive effect of thrombin was observed even in the absence of AngII stimulation as shown by the first point of the red curves in **Figures 1D, F**. In the case of BRET between AT1R-Rluc and Venus-Gαq, the curve almost reached its maximal level upon stimulation with thrombin (**Figure 1D**). By contrast, in AT1R-Rluc/yPET-β-arrestin 2 coexpressing cells, the co-stimulation with thrombin did not seem to affect significantly the AngII dose-response with no changes in AngII’s EC_50_ and E_max_ (**Figure 1F**). These observations demonstrate that thrombin leads to increase AT1R/Gαq coupling and AT1R/yPET-β-arrestin 2 interaction in PAR1-dependent manner suggesting a transactivation of AT1R by thrombin and PAR1.

**Figure 2:**
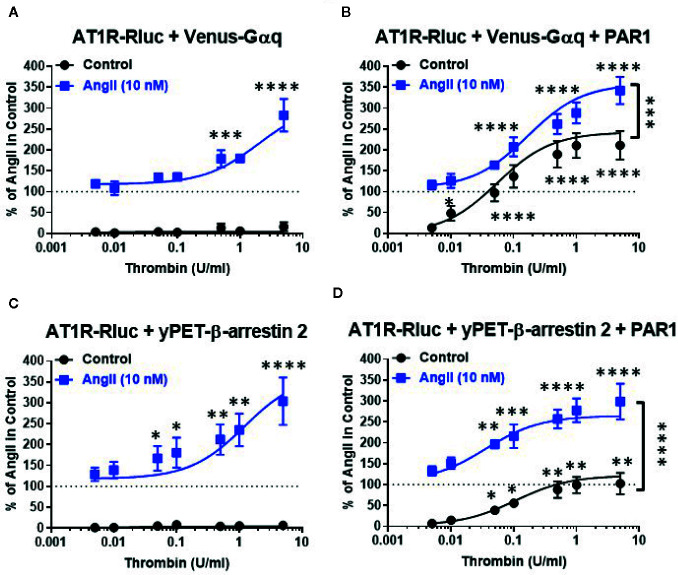
Dose-response BRET analysis of thrombin-induced AT1R/Gαq coupling and AT1R/β-arrestin 2 interaction. HEK293 cells transiently coexpressing AT1R-Rluc with either Venus-Gαq **(A, B)** or yPET-β-arrestin 2 **(C, D)** in the absence or presence of PAR1 overexpression as indicated were used. Cells were then treated 30 min at 37°C with the increasing concentrations of thrombin in the absence or presence of a sub-saturating dose of AngII (10 nM) before BRET signals were measured in live cells. Data are means ± SEM of three to four independent experiments performed in triplicate. The statistical analysis indicates the significance relative to the condition in the absence of thrombin for both curves, the control, and the ones in the presence of 10 nM of AngII. ****p-value < 0.0001, ***p-value < 0.001, **p-value < 0.01, *p-value < 0.05.

To further investigate such a positive interaction between AT1R and thrombin and its receptor PAR1, we also performed the dose-response analysis with thrombin on AT1R/Gαq and AT1R/β-arrestin 2 interactions in the absence and presence of PAR1 overexpression and AT1R co-activation (**Figure 2**). In the absence of PAR1 overexpression, thrombin-promoted BRET increase was neither observed in AT1R-Rluc/Venus-Gαq (**Figure 2A**) nor in AT1R-Rluc/yPET-β-arrestin 2 (**Figure 2C**) coexpressing cells whatever thrombin doses used. This completely ruled out any non-specific action of thrombin on AT1R in the absence of AngII stimulation as well as in the absence of PAR1 coexpression. In the absence of PAR1 overexpression, the co-treatment with 10 nM of AngII, corresponding to AngII’s EC_50_ dose as shown in **Figure 1**, however, resulted in a nice positive effect on thrombin dose curves. Indeed, AngII co-stimulation led to a strong potentiation in the thrombin-induced BRET signal up to 283 ± 39% and 304 ± 57% (*p-value <0.0001, n=4*) compared to AngII response alone (taken as 100%) in AT1R-Rluc/Venus-Gαq (**Figure 2A**) and AT1R-Rluc/yPET-β-arrestin 2 (**Figure 2C**) coexpressing cells, respectively. These data are consistent with the data in **Figure 1** suggesting a positive interaction between AngII and thrombin on ligand-promoted BRET increase between AT1R-Rluc/Venus-Gαq and AT1R-Rluc/yPET-β-arrestin 2 pairs.

In the condition of PAR1 overexpression, thrombin alone (without AngII cotreatment and AT1R activation) substantially promoted a dose-dependent BRET increase in both AT1R-Rluc/Venus-Gαq (E_max_ = 211 ± 34%, *p-value <0.0001, n=4*) (**Figure 2B**) and AT1R-Rluc/yPET-β-arrestin 2 (E_max_ = 102 ± 25%, *p-value <0.0001, n=3*) (**Figure 2D**) coexpressing cells compared to the signals induced by 10 nM of AngII alone (taken as 100%). It is worth noting that this was not observed in the absence of PAR1 coexpression (**Figures 2A, C**) indicating the specificity of thrombin-induced BRET responses. More importantly, under these conditions the maximal thrombin-dependent responses were strongly potentiated by the co-stimulation with 10 nM of AngII in both AT1R-Rluc/Venus-Gαq (E_max_ = 342 ± 33%, *p-value <0.0001, n=4*) (**Figure 2B**) and AT1R-Rluc/yPET-β-arrestin 2 (E_max_ = 299 ± 43%, *p-value <0.0001, n=3*) (**Figure 2D**) coexpressing cells. Together, these observations further demonstrate the positive interaction between AngII and thrombin regarding Gαq coupling and β-arrestin 2 recruitment and this occurred at the level of their respective receptors, AT1R and PAR1.

### Real-Time Kinetic Analysis of AT1R-PAR1 Interaction

**Figure 3:**
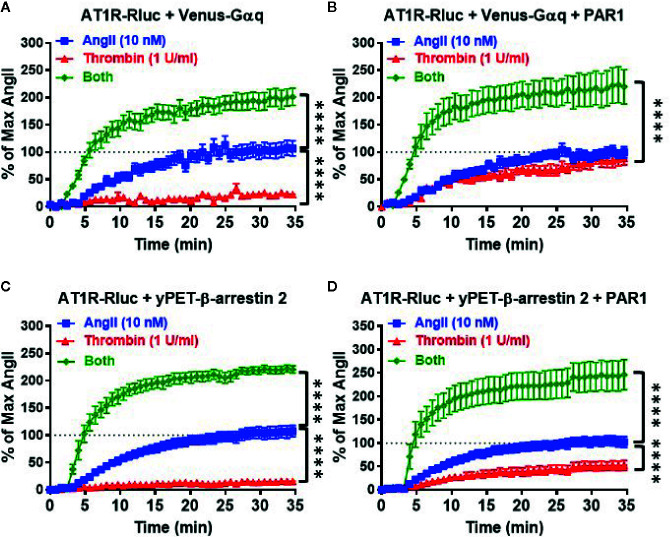
Real-time kinetic analysis of BRET for AT1R/Gαq coupling and AT1R/β-arrestin 2 interaction. HEK293 cells transiently coexpressing AT1R-Rluc with either Venus-Gαq **(A, B)** or yPET-β-arrestin 2 **(C, D)** in the absence or presence of PAR1 overexpression as indicated were used. Cells were then treated or not with either 10 nM of AngII, 1 U/ml of thrombin, or both, before BRET signals were measured in real-time and live cells for 35 min. Data are means ± SEM of 10–11 independent experiments performed in single-point measurements. The statistical analysis indicates the significance relative to the kinetic curves in the presence of 10 nM of AngII. ****p-value < 0.0001.

Next, we performed real-time kinetics on BRET signal increase in live cells coexpressing AT1R-Rluc with either Venus-Gαq (**Figures 3A, B**) or yPET-β-arrestin 2 (**Figures 3C, D**) in the absence or presence of PAR1 coexpression and upon stimulation with either 10 nM of AngII, 1 U/ml of thrombin, or both. In the absence of PAR1, AngII but not thrombin nicely promoted a significant time-dependent BRET increase between AT1R-Rluc/Venus-Gαq (**Figure 3A**) and AT1R-Rluc/yPET-β-arrestin 2 (**Figure 3C**) pairs. Interestingly, under these conditions of no PAR1 overexpression, the co-stimulation of cells with both AngII and thrombin strongly potentiated the BRET increase up to 205 ± 3% of AngII response (*p-value <0.0001, n=11*) in AT1R-Rluc/Venus-Gαq (**Figure 3A**) and up to 214 ± 2% of AngII response (*p-value <0.0001, n=10*) in AT1R-Rluc/yPET-β-arrestin 2 (**Figure 3C**) coexpressing cells. These observations suggest a positive allosteric effect of thrombin on AngII and further demonstrate the positive interaction between thrombin and AngII concerning AT1R-Gαq coupling and β-arrestin 2 recruitment to AT1R, which may involve thrombin receptors endogenously expressed in HEK293 cells.

In the presence of PAR1 overexpression, as expected thrombin promoted a nice but partial time-dependent BRET increase between AT1R-Rluc/Venus-Gαq (E_max_ = 79 ± 2% of AngII response, *p-value <0.0001, n=11*) (**Figure 3B**) and AT1R-Rluc/yPET-β-arrestin 2 (E_max_ = 44 ± 2% of AngII response, *p-value <0.0001, n=11*) (**Figure 3D**) pairs. Furthermore, the concomitant stimulation of the cells with AngII and thrombin also resulted in a strong potentiation of the BRET signals between AT1R-Rluc/Venus-Gαq (E_max_ = 207 ± 4% of AngII response, *p-value <0.0001, n=11*) (**Figure 3B**) and AT1R-Rluc/yPET-β-arrestin 2 (E_max_ = 218 ± 4% of AngII response, *p-value <0.0001, n=10*) (**Figure 3D**) pairs compared to the single treatments with AngII and thrombin. Such a potentiation was more than additive suggesting the synergistic interaction between AngII and thrombin at the level of their receptors. These observations demonstrated that the combination of AngII and thrombin was more efficient to elicit BRET increase within AT1R-Rluc/Venus-Gαq and AT1R-Rluc/yPET-β-arrestin 2 pairs than the individual activation of both AT1R or PAR1. This further supports the synergistic and positive allosteric interaction between AT1R and PAR1.

**Figure 4:**
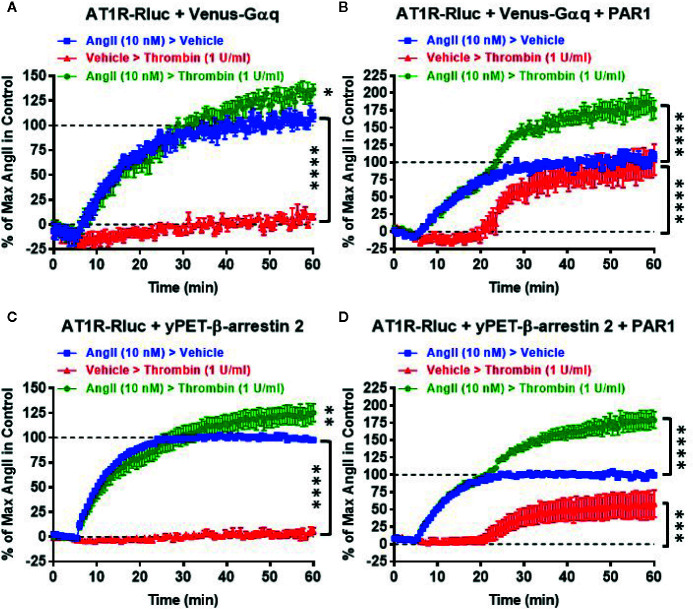
Sequential real-time kinetic analysis of BRET for AT1R/Gαq coupling and AT1R/β-arrestin 2 interaction. HEK293 cells transiently coexpressing AT1R-Rluc with either Venus-Gαq **(A, B)** or yPET-β-arrestin 2 **(C, D)** in the absence or presence of PAR1 overexpression as indicated were used. Cells were then sequentially treated or not (vehicle) with either 10 nM of AngII, 1 U/ml of thrombin, or both, as indicated. BRET signals were then measured in real-time and live cells for 60 min. Data are means ± SEM of eight to nine independent experiments performed in single-point measurements. The statistical analysis indicates the significance relative to the baseline as well as to the kinetic curves in the presence of 10 nM of AngII. ****p-value < 0.0001, ***p-value < 0.001, **p-value < 0.01, *p-value < 0.05.

Next, we also performed real-time kinetics with the sequential treatments with vehicle (used as a negative control), AngII (10 nM), and thrombin (1 U/ml) of live cells coexpressing AT1R-Rluc with either Venus-Gαq (**Figures 4A, B**) or yPET-β-arrestin 2 (**Figures 4C, D**) in the absence or presence of PAR1 coexpression. This aimed to reveal the synergistic and the positive allosteric interaction between AngII and thrombin at the level of their receptors. For this, after recording the basal BRET during 5 min cells were first stimulated with either vehicle or AngII (treatment 1 or T1), and BRET signals were measured for 15 min to promote AT1R-Rluc/Venus-Gαq and AT1R-Rluc/yPET-β-arrestin 2 interaction as indicated in **Figure 4**. Then, a second treatment (treatment 2 or T2) was applied by stimulating cells with either vehicle or thrombin and BRET signals were measured for up to 40 min. In all the conditions, the treatment of cells with AngII triggered a time-dependent BRET increase between AT1R-Rluc/Venus-Gαq and AT1R-Rluc/yPET-β-arrestin 2 (**Figure 4**).

In the absence of PAR1 coexpression, as expected the sequential treatment with a vehicle followed by thrombin did not promote any significant time-dependent BRET increase in both AT1R-Rluc/Venus-Gαq (**Figure 4A**) and AT1R-Rluc/yPET-β-arrestin 2 (**Figure 4C**) expressing cells. Moreover, the sequential treatment with AngII followed by thrombin showed a weak but significant effect on AngII-induced response on AT1R activation (**Figures 4A, C**) compared to the treatment with AngII followed by vehicle. However, upon PAR1 overexpression, while vehicle treatment had no significant effect on BRET signals the sequential addition of thrombin elicited a nice time-dependent BRET increase between AT1R-Rluc/Venus-Gαq (**Figure 4B**) and AT1R-Rluc/yPET-β-arrestin 2 (**Figure 4D**) pairs with a full effect on AT1R-Rluc/Venus-Gαq coupling (**Figure 4B**) and a partial effect (∼50% of AngII response) on AT1R-Rluc/yPET-β-arrestin 2 interaction (**Figure 4D**). Interestingly, the sequential treatment of cells with AngII until reaching the plateau then followed by thrombin substantially raised the BRET responses significantly beyond the responses mediated by AngII or thrombin applied alone (**Figures 4B, D**). These observations indicated that the positive interaction between AngII and thrombin can occur sequentially. Moreover, the combination of AngII and thrombin was more efficient to elicit BRET increase within AT1R-Rluc/Venus-Gαq and AT1R-Rluc/yPET-β-arrestin 2 pairs than the individual activation of both AT1R or PAR1. This confirms again the positive interaction between AngII and thrombin and further supports the synergistic and positive allosteric interaction between AT1R and PAR1.

### The Positive Functional AT1R-PAR1 Interaction Occurred in Receptor Expression-Dependent Manner

**Figure 5:**
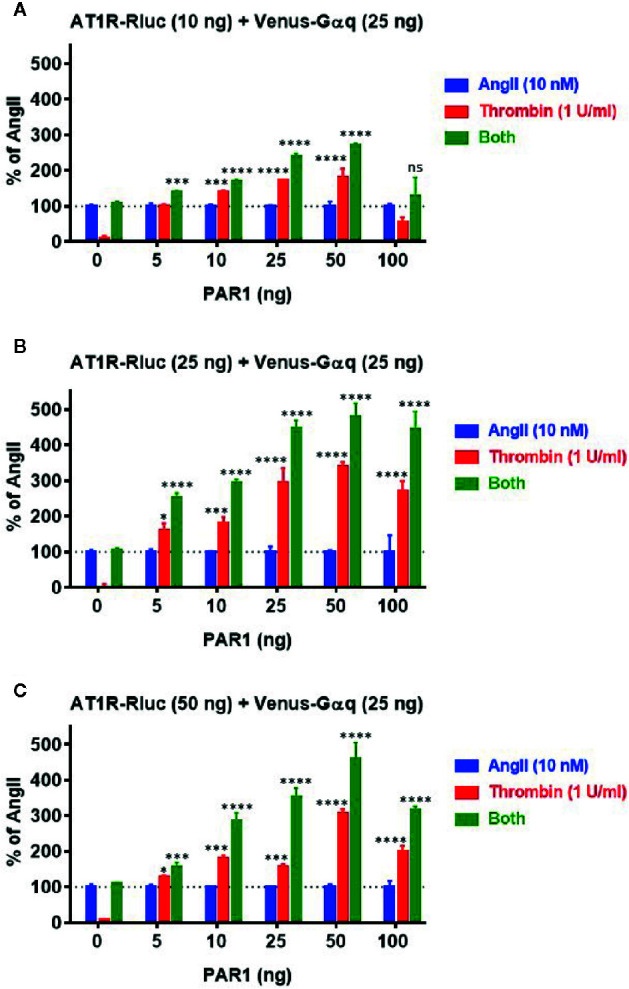
BRET titration experiments for AT1R/Gαq coupling. HEK293 cells were transiently transfected with 25 ng of Venus-Gαq plasmid (as BRET acceptor) and different amount of AT1R-Rluc plasmid (as BRET donor): 10 ng **(A)**, 25 ng **(B)**, and 50 ng **(C)**, in the absence (0 ng) or presence of increasing amounts of PAR1 plasmid (5, 10, 25, 50, and 100 ng). Cells were then stimulated or not with either 10 nM of AngII, 1 U/ml of thrombin, or both, for 30 min at 37°C, before BRET signals were measured. Data are means ± SEM of three independent experiments performed in triplicate. The statistical analysis indicates the significance relative to the response of AngII. ****p-value < 0.0001, ***p-value < 0.001, *p-value < 0.05, and ns p-value > 0.05.

Next, we profiled the positive functional interaction between AT1R and PAR1 and their synergy in BRET assays using various transient expression ratios between the receptors. For this, BRET titration assays were performed using three different amounts of AT1R-Rluc (BRET donor): 10 ng (**Figure 5A**), 25 ng (**Figure 5B**), and 50 ng (**Figure 5C**), without or with increasing amounts of PAR1 (5 ng, 10 ng, 25 ng, 50 ng, and 100 ng) and a constant amount (25 ng) of Venus-Gαq (BRET acceptor). All the cells were then treated or not with either AngII (10 nM), thrombin (1 U/ml), or both. The overall ligand-induced BRET increase between AT1R-Rluc and Venus-Gαq was tightly correlated with the expression levels of both AT1R-Rluc and PAR1 showing better responses with 25 ng (**Figure 5B**) or 50 ng (**Figure 5C**) compared to 10 ng (**Figure 5A**) of AT1R-Rluc. Moreover, in the absence of PAR1 coexpression thrombin alone did not promote any BRET increase while AngII nicely did and no significant BRET potentiation was observed upon co-stimulation with AngII and thrombin. However, thrombin-dependent BRET responses significantly increased with the increase of PAR1 expression until reaching saturated levels from 25 ng of PAR1 and this regardless of the amount of AT1R-Rluc used. Importantly, the increase in PAR1 coexpression was also associated with a strong and a saturated BRET potentiation upon combined treatment of the cells with AngII and thrombin. Such a correlation was more significant with 25 ng (**Figure 5B**) and 50 ng (**Figure 5C**) of AT1R-Rluc but was also observed even with very low expression (5 to 10 ng) of either AT1R-Rluc or PAR1. Together, these BRET titration experiments demonstrate that thrombin-induced BRET increase between AT1R-Rluc and Venus-Gαq and its potentiation by the combined AngII/thrombin treatment can be observed at very low expression levels of the receptors. Moreover, this was nicely correlated with the amount of PAR1 coexpressed and saturated at a certain level of expression indicating the specificity of thrombin/PAR1-mediated BRET within AT1R/Gαq pair. Based on these BRET titration data all our BRET experiments were carried out by taking 25 ng of AT1R-Rluc, 25 ng of Venus-Gαq, and 25 ng of PAR1 (**Figure 5B**) as the most appropriate transfection condition.

### Effect of Receptor Blockade on AT1R Transactivation by PAR1

**Figure 6:**
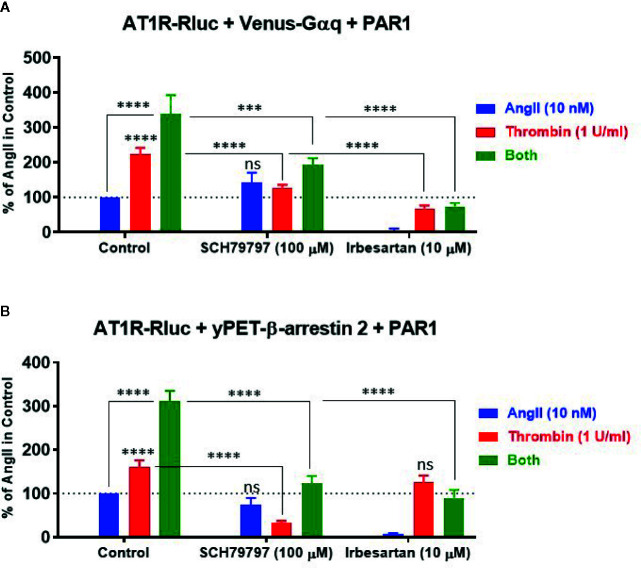
Effect of receptor blockade on AT1R/Gαq coupling and AT1R/β-arrestin 2 interaction assessed by BRET. HEK293 cells transiently coexpressing AT1R-Rluc with either Venus-Gαq **(A)** or yPET-β-arrestin 2 **(B)** in the presence of PAR1 overexpression were used. Cells were first pretreated or not (control) with 100 µM of SCH79797 (PAR1 antagonist) or 10 µM of irbesartan (AT1R antagonist) as indicated, for 15 min at 37°C. Cells were then stimulated or not with either 10 nM of AngII, 1 U/ml of thrombin, or both, for 30 min at 37°C, before BRET signals were measured in live cells. Data are means ± SEM of four to six independent experiments performed in triplicate. The statistical analysis indicates the significance relative to the response of AngII or thrombin alone as well as to the condition of the combined treatment in control as indicated. ****p-value < 0.0001, ***p-value < 0.001, and ns p-value > 0.05.

To characterize further the pharmacological action of thrombin and its receptor PAR1 on AT1R and their functional interaction, we examined the effect of receptor blockade on AngII- and thrombin-induced BRET signals between AT1R-Rluc/Venus-Gαq (**Figure 6A**) and AT1R-Rluc/yPET-β-arrestin 2 (**Figure 6B**) pairs in the presence of PAR1 overexpression. For this, we used irbesartan at 10 µM as AT1R-selective antagonist and SCH79797 dihydrochloride at 100 µM as PAR1-selective antagonist. As expected, irbesartan fully blocked AngII-mediated responses in both AT1R-Rluc/Venus-Gαq (**Figure 6A**) and AT1R-Rluc/yPET-β-arrestin 2 (**Figure 6B**) expressing cells (*p-value <0.0001, n=6*). Interestingly, irbesartan also strongly diminished thrombin-mediated BRET signal in AT1R-Rluc/Venus-Gαq (*p-value <0.0001, n=6*) (**Figure 6A**) but not in AT1R-Rluc/yPET-β-arrestin 2 (**Figure 6B**) expressing cells. This suggests that part of thrombin-promoted AT1R-Rluc/Venus-Gαq coupling involves AT1R activation perhaps *via* its transactivation by PAR1 as suggested above. On the other hand, PAR1 antagonist (SCH79797) significantly diminished to different extent thrombin-promoted BRET signals in AT1R-Rluc/Venus-Gαq (*p-value <0.0001, n=4*) (**Figure 6A**) and AT1R-Rluc/yPET-β-arrestin 2 (*p-value <0.0001, n=4*) (**Figure 6B**) expressing cells. However, in both cases, SCH79797 did not affect the signals elicited by AngII treatment indicating the specificity of the effects. In the condition of the combined treatment with AngII and thrombin, both irbesartan and SCH79797 drastically decreased the BRET signals and fully abolished the potentiating effect of the cotreatment for both AT1R-Rluc/Venus-Gαq (**Figure 6A**) and AT1R-Rluc/yPET-β-arrestin 2 (**Figure 6B**) pairs (*p-value <0.0001, n=4-6*). This demonstrates the implication of the activation of both, AT1R and PAR1, in the synergistic and positive interaction between thrombin and AngII.

### Effect of AT1R-PAR1 Interaction on the Gαq/Inositol Phosphate Signaling Pathway

**Figure 7:**
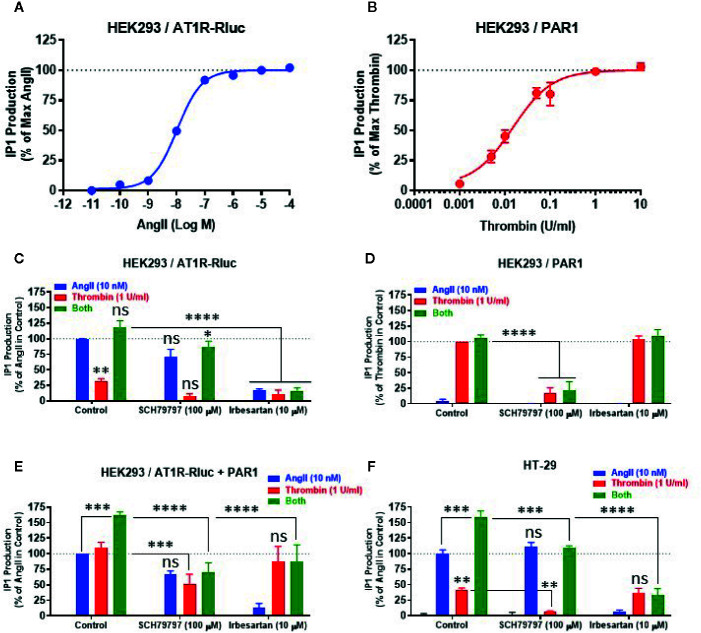
Effect of receptor blockade on IP1 production. HEK293 cells transiently expressing either AT1R-Rluc **(A, C)**, PAR1 **(B, D)**, or both **(E)**, and HT-29 cells **(F)** were first pretreated or not (control) with 100 µM of SCH79797 or 10 µM of irbesartan as indicated, for 15 min at 37°C. Cells were then stimulated or not with either increasing doses of AngII or thrombin **(A, B)**, 10 nM of AngII, 1 U/ml of thrombin, or both **(C–F)**, for 30 min at 37°C, before IP1 production was quantified. Data are means ± SEM of three (for dose-response in HEK293 and antagonist data in HT-29) or four (for antagonist data in HEK293) independent experiments performed in triplicate. The statistical analysis indicates the significance relative to the response of AngII or thrombin alone as well as to the condition of the combined treatment in control as indicated. ****p-value < 0.0001, ***p-value < 0.001, **p-value < 0.01, *p-value < 0.05, and ns p-value > 0.05.

To correlate the BRET data observed between AT1R-Rluc and Venus-Gαq with the intracellular downstream signaling pathway, we examined the effect of AT1R and PAR1 coexpression and their activation on Gαq/inositol phosphate (IP1) pathway by measuring the accumulation of IP1 in live cells. First, we performed the dose-response analysis in HEK293 expressing AT1R-Rluc (**Figure 7A**) or PAR1 (**Figure 7B**) to check whether the concentrations of AngII (EC_50_) and thrombin (saturating) to be used in the IP1 assay could be similar to what was used before in BRET assays. Indeed, the saturating IP1 dose-curves with AngII and thrombin on their respective receptors shown in **Figures 7A, B** are consistent with those obtained in BRET assay (**Figures 1A, B**). They clearly showed that 10 nM corresponds pretty well to the EC_50_ of AngII on AT1R-mediated IP1 production (**Figure 7A**), whereas 1 U/ml largely constitutes the saturating dose of thrombin on PAR1-mediated IP1 response (**Figure 7B**). Then a full set of experiments was carried out in HEK293 cells expressing either AT1R-Rluc (**Figure 7C**), PAR1 (**Figure 7D**), or AT1R-Rluc and PAR1 (**Figure 7E**) were first pretreated or not with SCH79797 (100 μM) or irbesartan (10 μM) before their stimulation with either AngII (10 nM), thrombin (1 U/ml), or their combination, as indicated. As shown in **Figure 7C**, in AT1R-Rluc expressing cells AngII, but not thrombin, induced IP1 production that was fully blocked by irbesartan but not SCH79797. In these cells, a partial thrombin-mediated IP1 response was also observed reflecting endogenous expression of thrombin receptors as stated above. By contrast, in cells overexpressing PAR1 thrombin but not AngII induced IP1 production that was blocked by SCH79797 but not irbesartan (**Figure 7D**). These data demonstrate the specificity of the pharmacological blockade of the receptors and are consistent with our dose-response BRET curves shown in **Figures 1A, B** confirming the functional coupling of AT1R and PAR1 to Gαq/inositol phosphate pathway.

In cells coexpressing AT1R-Rluc and PAR1, in addition to the IP1 response promoted by the individual treatment with AngII or thrombin, we also observed a synergistic effect (∼160%) upon their combined treatment (**Figure 7E**). This is consistent with our BRET observations and further demonstrates the synergistic functional interaction between AT1R and PAR1 for the Gαq/inositol phosphate pathway. More interestingly, both SCH79797 and irbesartan fully inhibited the potentiating effect (green bars) of the combined treatment (**Figure 7E**) further indicating the implication of the activation of both AT1R and PAR1 in the synergistic and positive interaction between thrombin and AngII at their downstream signaling. Moreover, the data indicate that the blockade of one receptor did not fully impair the IP1 response which may be due to the symmetrical coupling to Gαq of AT1R and PAR1 protomers within AT1R-PAR1 complex or to their monomer/homomer receptor populations coexistent in HEK293 cells.

Next, we also investigated the impact of AT1R-PAR1 functional interaction on the Gαq/inositol phosphate pathway in the human epithelial colorectal adenocarcinoma cells (HT-29) previously reported to express endogenous AT1R (Ozeki et al., 2013; Wang et al., 2013) and PAR1 (Darmoul et al., 2004). As shown in **Figure 7F**, the overall IP1 response pattern was similar to what was obtained in HEK293 cells coexpressing AT1R-Rluc and PAR1 (**Figure 7E**). Indeed, both AngII and thrombin showed a positive IP1 response that was specifically blocked by irbesartan and SCH79797, respectively (**Figure 7F**). Moreover, the combination of AngII and thrombin elicited a significant potentiation of the IP1 response which was either drastically or only partially blocked by irbesartan or SCH79797, respectively (**Figure 7F**). Together, these IP1 data in HT-29 confirm the reality of the positive functional interaction between endogenously expressed AT1R and PAR1 receptors and further demonstrate the implication of their dual activation in the synergistic and positive interaction between thrombin and AngII at their downstream signaling.

### Thrombin and PAR1 Activation Negatively Modulates AT1R Internalization and Its Endosomal Trafficking

**Figure 8:**
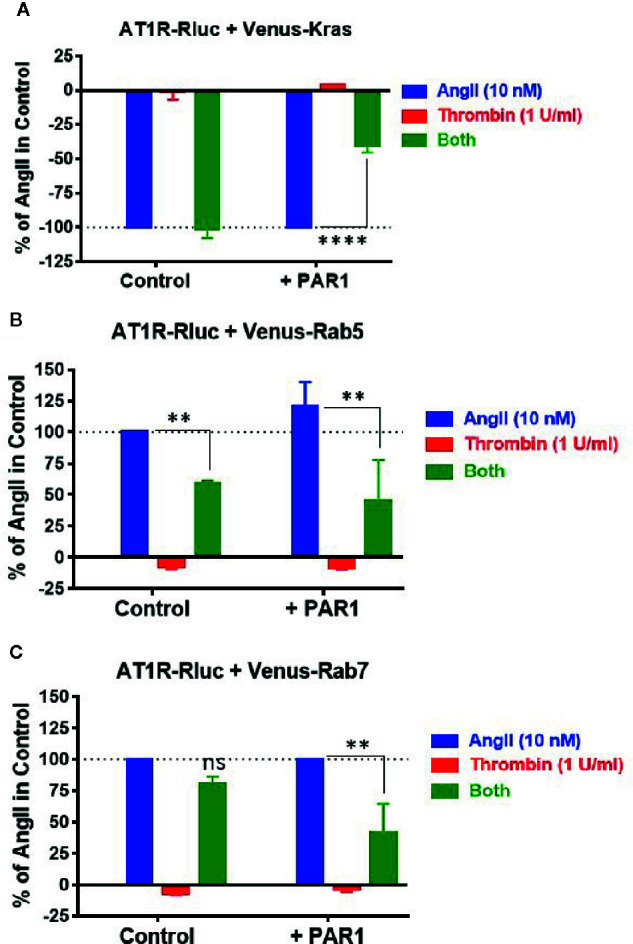
Internalization and endosomal trafficking of AT1R assessed by BRET assays. HEK293 cells transiently coexpressing AT1R-Rluc with either Venus-Kras **(A)**, Venus-Rab5 **(B)**, or Venus-Rab7 **(C)** in the absence or presence of PAR1 overexpression were used. Cells were then stimulated or not with either 10 nM of AngII, 1 U/ml of thrombin, or both, for 30 min at 37°C, before BRET signals were measured in live cells. Data are means ± SEM of three independent experiments performed in triplicate. The statistical analysis indicates the significance relative to the condition in the presence of 10 nM of AngII. ****p-value < 0.0001, **p-value < 0.01, and ns p-value > 0.05.

Finally, we investigated the other important aspect of GPCR function and regulation which is internalization and intracellular trafficking. Indeed, the effect of thrombin and PAR1 overexpression and activation on AT1R internalization and endosomal trafficking were examined using specific BRET sensors, Venus-Kras and Venus-Rabs, respectively, as previously reported (Lan et al., 2011; Lan et al., 2012; Ayoub et al., 2015a). For the internalization, AngII (10 nM) but not thrombin (1 U/ml) induced AT1R internalization as illustrated by a nice decrease in the BRET signal in cells coexpressing AT1R-Rluc and Venus-Kras (**Figure 8A**). Such AngII-mediated response was not affected by the co-stimulation of cells with thrombin and even by the simple coexpression of PAR1 (**Figure 8A**). However, in cells coexpressing AT1R-Rluc, Venus-Kras, and PAR1, the simultaneous activation with AngII and thrombin significantly diminished AT1R internalization by ∼60% (E_max_ = -41 ± 5%, *p-value <0.0001, n=3*) (**Figure 8A**). Of course, this effect was specific to PAR1 since it was not observed in its absence as shown in **Figure 8A**. Moreover, this observation on AT1R internalization was supported by those obtained on its intracellular endosomal trafficking using the small GTPase, Rab5, and Rab7, as specific markers for the early and late endosomes, respectively. For this, BRET signals were measured in cells coexpressing AT1R-Rluc with either Venus-Rab5 (**Figure 8B**) or Venus-Rab7 (**Figure 8C**) where AngII nicely increased the BRET signals between AT1R-Rluc and Venus-Rab5/7 indicating AT1R trafficking in the early and then late endosomes following its internalization. Interestingly, PAR1 overexpression and co-activation significantly decreased the BRET signals with both Rab sensors (E_max_ = 45 ± 32% of AngII alone (120 ± 20%) for Rab5, and E_max_ = 41 ± 23% for Rab7, *p-value <0.01, n=3*), so AT1R endosomal trafficking (**Figures 8B, C**). The significant effect of the combined treatment with AngII and thrombin observed with Rab5 (E_max_ = 59 ± 2%*, p-value <0.01, n=3*) and to less extent Rab7 (E_max_ = 81 ± 6%*, p-value *<* 0.1, n=3*) in the absence of PAR1 coexpression may due to the endogenous thrombin receptors expressed in HEK293 as observed in BRET assays with Gαq and β-arrestin (**Figure 1**). These observations are consistent with the BRET data obtained on AT1R internalization (**Figure 8A**) further indicating the inhibitory action of thrombin and PAR1 overexpression and activation on AngII-mediated AT1R internalization and endosomal trafficking.

### The Functional AT1R-PAR1 Interaction Implies Their Physical Interaction in HEK293 Cells

**Figure 9:**
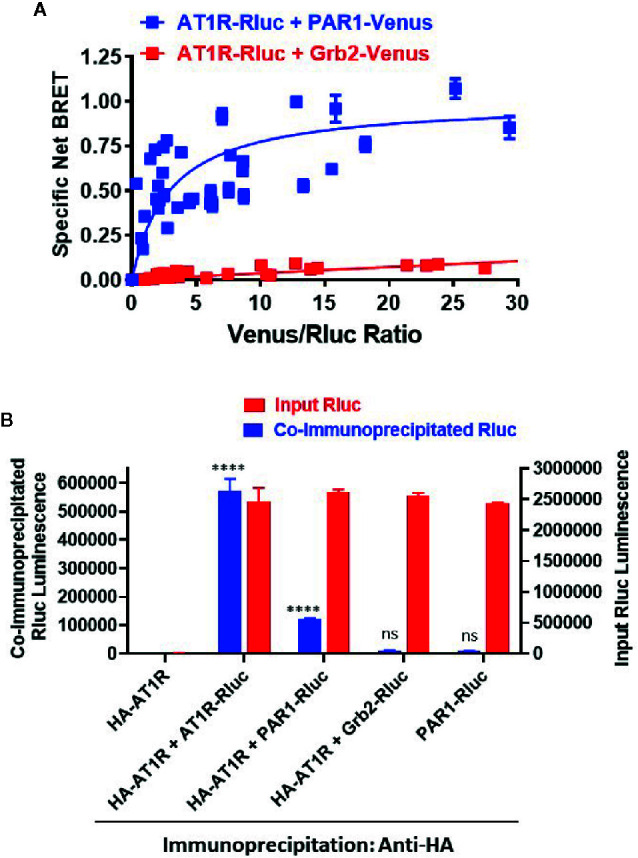
Physical interaction between AT1R and PAR1 studied by BRET saturation in live cells and co-immunoprecipitation. **(A).** HEK293 cells transiently coexpressing a constant amount of AT1R-Rluc (BRET donor) without or with the increasing amount of either PAR1-Venus or Grb2-Venus (BRET acceptors) were used for BRET saturation in live cells. The fluorescence of Venus-tagged proteins, the luminescence of AT1R-Rluc, and the BRET signals were measured in parallel at room temperature. Then, the net BRET signals were plotted over the fluorescence/luminescence (Venus/Rluc) ratios. **(B)**. HEK293 cells transiently coexpressing HA-AT1R without or with either AT1R-Rluc, PAR1-Venus, or Grb2-Venus were used for co-immunoprecipitation using the anti-tag HA antibody. For this, an equal Rluc input was used and the final immunoprecipitates were then used for the quantification of Rluc luminescence of the different Rluc-tagged proteins co-immunoprecipitated or not with HA-AT1R (co-immunoprecipitated Rluc). The BRET saturation data are means ± SEM of three to four independent experiments performed in triplicate. The co-immunoprecipitation data are means ± SEM of three independent measurements. ****p-value < 0.0001 and ns p-value > 0.05.

The overall BRET data with Gαq and β-arrestin indicate a proximity between AT1R and PAR1 suggesting their possible physical interaction when co-expressed in HEK293 cells. To investigate this aspect we used the classical BRET saturation assay (Ayoub and Pfleger, 2010; Ayoub, 2016) as well as co-immunoprecipitation between differentially tagged receptors. For BRET saturation, a constant amount of PAR1-Rluc (as BRET donor) was co-expressed with the increasing amount of either PAR1-Venus (BRET acceptor) or the adaptor signaling protein, Grb2-Venus (as a negative control). As shown in **Figure 9A**, the net BRET signal plotted over the Venus/Rluc ratio nicely increased with PAR1-Venus but not Grb2-Venus until reaching the saturation demonstrating the specificity of the BRET signal occurred between AT1R-Rluc and PAR1-Venus and suggesting their existence in proximity. To further support this conclusion, we also performed co-immunoprecipitation experiments between HA-tagged AT1R and PAR1-Rluc where HA-AT1R was immunoprecipitated using the anti-tag HA antibody followed by measurements of Rluc luminescence specific to PAR1-Rluc co-immunoprecipitated with HA-AT1R. For this, HEK293 coexpressing HA-AT1R without or with either PAR1-Rluc, AT1R-Rluc as a positive control for HA-AT1R/AT1R-Rluc homodimers, or Grb2-Rluc as a negative control, were used (**Figure 9B**). As shown in **Figure 9B**, for a similar input of Rluc-tagged proteins used from all the samples a strong luminescence signal was measured in the immunoprecipitate obtained from HA-AT1R/AT1R-Rluc expressing cells demonstrating their physical interaction and homodimerization as previously reported (Hansen et al., 2004; Porrello et al., 2011; Young et al., 2017) and validating our assay. Moreover, a weaker but a significant Rluc signal was also measured in the immunoprecipitate obtained from HA-AT1R/PAR1-Rluc expressing cells (**Figure 9B**). Such a signal was observed neither when PAR1-Rluc was expressed alone without HA-AT1R nor in the immunoprecipitate obtained from HA-AT1R/Grb2-Rluc expressing cells indicating the specificity of the co-immunoprecipitation (**Figure 9B**). These data support the physical interaction between AT1R and PAR1 when coexpressed in HEK293 cells that may explain their positive and synergistic functional interaction.

## Discussion

In this study, we investigated the functional and pharmacological effect of thrombin and its receptor PAR1 on AT1R activity, signaling, and regulation when they are transiently coexpressed in HEK293 cells using various BRET-based proximity assays. The rationale behind our *in vitro* study came from many previous *in vitro* and *in vitro* studies demonstrating the link between RAS, AngII, and thrombin, in the vascular system (Weber, 1994; Polevaya, 1996; Kim and Iwao, 2011; Hasan et al., 2019). However, the implication of AngII and thrombin receptors in such interplay was not fully understood. Therefore, we hypothesized that the interplay between AngII and thrombin may occur at the level of their specific receptors in the different target cells and tissues. To test this, we examined one aspect of such functional interplay by investigating the pharmacological action of thrombin and its receptor PAR1 on AT1R function in HEK293 cells. Our approach was based on various BRET-based proximity assays inspired by the GPCR-heteromer investigation technology (GPCR-HIT) previously used to study GPCR and receptor tyrosine kinase (RTK) heteromers (Johnstone and Pfleger, 2012; Mustafa et al., 2012; Ayoub et al., 2013; Ayoub et al., 2015b). Moreover, such a functional interplay at the level of receptors was correlated with the intracellular downstream Gαq/inositol phosphate signaling as well as with the putative physical interaction between AT1R and PAR1.

**Figure 10:**
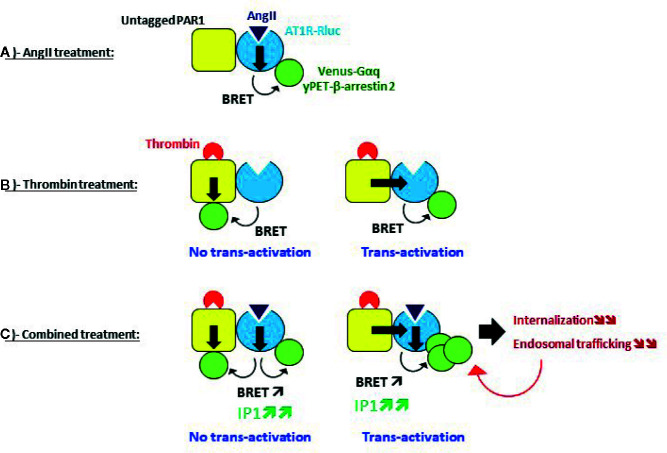
Speculative model of AT1R-PAR1 interplay and its effect on AT1R activity and regulation revealed by BRET. **(A)** Single AT1R activation, **(B)** Single PAR1 activation, and **(C)** Dual activation of AT1R and PAR1.

Our first main finding is that thrombin through PAR1 activation was able to promote BRET increase within AT1R-Rluc and Venus-Gαq or yPET-β-arrestin 2 pairs reflecting AT1R activation. This suggests the transactivation of AT1R by thrombin and PAR1 which specifically occurred in a thrombin dose-dependent manner and cells overexpressing PAR1 in a receptor expression dependent manner and demonstrated by both dose-response and real-time kinetic BRET analysis. Indeed, since thrombin did not directly act on AT1R and due to the proximity-based feature of the different BRET assays used, our data support the possible transactivation of AT1R-Rluc by thrombin and PAR1 and an asymmetrical recruitment of Venus-Gαq and yPET-β-arrestin 2 to AT1R-Rluc as illustrated in **Figure 10B** and as reported for other GPCR pairs using similar approaches (Mustafa et al., 2012; Ayoub et al., 2013; Ayoub et al., 2015b). The inhibitory effect of PAR1 antagonist (SCH79797) on thrombin-promoted BRET signal within AT1R-Rluc/Venus-Gαq and AT1R-Rluc/yPET-β-arrestin 2 pairs (**Figure 6**) is consistent with PAR1 activation being required for such a transactivation of AT1R by thrombin. The remaining response may be due to the fraction of PAR1 not fully inhibited by SCH79797 or the implication of other thrombin receptors that might not be sensitive to SCH79797 and endogenously expressed in HEK293 such as PAR4 (Schmidt et al., 1996; Atwood et al., 2011).

Alternatively, thrombin-promoted BRET increase between AT1R-Rluc and Venus-Gαq or yPET-β-arrestin 2 could simply due to the asymmetrical PAR1/Venus-Gαq coupling and PAR1/yPET-β-arrestin 2 interaction along with that directly occurred with AT1R-Rluc (**Figure 10B**). Such a possibility was supported by the data using the AT1R-selective antagonist (irbesartan) which did block AngII- but not thrombin-promoted BRET signal between AT1R-Rluc and yPET-β-arrestin 2 (**Figure 6B**). This suggests that thrombin-promoted AT1R/β-arrestin 2 interaction does not require the active conformation of AT1R and the BRET increase may reflect the recruitment of yPET-β-arrestin 2 (as BRET acceptors) directly to PAR1 (untagged) protomer bringing yPET-β-arrestin 2 in the vicinity of AT1R-Rluc (as BRET donor) for BRETsignal increase to occur. More interestingly, irbesartan strongly inhibited thrombin-induced BRET between AT1R-Rluc and Venus-Gαq (**Figure 6A**) but not between AT1R-Rluc and yPET-β-arrestin 2 (**Figure 6B**). This suggests that AT1R activation was required for thrombin/PAR1-mediated BRET response which could be consistent with the specific transactivation of AT1R/Gαq coupling but not AT1R/β-arrestin 2 interaction. However, this was not observed on Gαq/IP1 pathway since irbesartan did not significantly inhibit thrombin-dependent IP1 responses in HEK293 (**Figure 7C**) or HT-29 (**Figure 7D**) cells. In all cases, transactivation or not, only the physical proximity (direct or indirect) between AT1R-Rluc and PAR1 could lead BRET increase to occur between AT1R-Rluc and Venus-Gαq or yPET-β-arrestin 2 that explains thrombin-induced BRET increase (**Figure 10B**). Indeed, our BRET saturation assay in intact cells and *in vitro* co-immunoprecipitation data supported the direct physical interaction between AT1R and PAR1 in HEK293 cells that may explain their positive functional interaction. However, this aspect needs an in-depth investigation to demonstrate its reality and relevance in native tissues and a more integrated system. Thus, our data reveal for the first time the possible interplay between AT1R and PAR1 when coexpressed in HEK293 cells. This comes to extend the list of GPCR members potentially interacting or interplaying with AT1R (Takezako et al., 2017; Tóth et al., 2018) or PAR1 (Arachiche et al., 2013; Lin and Trejo, 2013) reported in many contexts and models.

The other important observation was obtained from the combined treatment with AngII and thrombin in AT1R/PAR1 coexpressing cells and also in the absence of PAR1 overexpression (**Figures 1C, E** and **3A, C**). Indeed, such combined treatment led to a substantially different profile in terms of BRET signals between AT1R-Rluc/Venus-Gαq and AT1R-Rluc/yPET-β-arrestin 2 pairs compared to the respective individual treatments with AngII and thrombin. This was observed with a non-saturating dose of AngII (10 nM) and a saturating dose of thrombin (1 U/ml). In the dose-response BRET experiments, the saturating dose of thrombin (1 U/ml) significantly left-shifted the AngII dose curve and increased its potency and this was mainly observed in the absence of PAR1 overexpression most likely involving other endogenous thrombin receptors since HEK293 were reported to predominantly express PAR1, PAR2 and to less extent PAR4 but only very low PAR3 (Schmidt et al., 1996; Atwood et al., 2011). By contrast, thrombin combination had no significant effect on the maximal AngII-induced BRET signals since the system seems to be fully saturated. Such an effect of thrombin suggests the putative positive allosteric modulation of AT1R by thrombin through endogenously expressed receptors in HEK293 cells. On the other hand, the cotreatment with AngII at its EC_50_ (10 nM) positively affected thrombin dose curves by increasing thrombin efficacy as illustrated by BRET potentiation within AT1R-Rluc/Venus-Gαq and AT1R-Rluc/yPET-β-arrestin 2 pairs to levels stronger than the maximal signals promoted by thrombin alone. The positive effects observed in dose-response experiments were also confirmed by the real-time kinetic analysis with the combined AngII/thrombin treatment. Indeed, in the absence of PAR1 overexpression, while thrombin did not affect the BRET signals, its combination strongly potentiated AngII-mediated BRET increase (**Figures 3A, C**) indicating a positive allosteric action of thrombin probably through its endogenously expressed receptors (PAR1 and/or PAR4) in HEK293 cells. This is consistent with the well-established link between the concept of allostery and the interactions between GPCRs highlighting the allosteric modulation between various GPCRs (Springael et al., 2007; Haack and McCarty, 2011; Gomes et al., 2016) such as GABAb heterodimer as well as heterodimers involving glutamate receptors (Pin and Bettler, 2016). Moreover, our data revealed that the activation of both receptors, AT1R and PAR1, was more efficient than their single activation with BRET effects that were even more than additive. This was observed in BRET within AT1R-Rluc/Venus-Gαq and AT1R-Rluc/yPET-β-arrestin 2 pairs as well as on Gαq/IP1 pathway in both HEK293 cells transiently coexpressing AT1R and PAR1 and in the human epithelial colorectal adenocarcinoma cells (HT-29) reported to express endogenous AT1R (Ozeki et al., 2013; Wang et al., 2013) and PAR1 (Darmoul et al., 2004). In HT-29 cells the data suggest the predominant implication of AT1R protomer in the potentiation of IP1 response since irbesartan almost fully inhibited the response (**Figure 7F**). Together, these data demonstrate that the positive functional interaction between AT1R and PAR1 observed at the level of receptor activation by BRET was nicely correlated with their intracellular downstream signaling pathway.

In **Figure 10C**, we speculated about the potentiating effect of the combination treatment with AngII and thrombin at the level of AT1R and PAR1 being in physical proximity, whether this implies the transactivation of AT1R by PAR1 or not. In case no transactivation occurred from PAR1 to AT1R, the potentiated BRET signals induced by AngII/thrombin combined treatment may be explained by symmetrical recruitment and association of Venus-Gαq and yPET-β-arrestin 2 with both AT1R and PAR1 protomers (**Figure 10C**). Accordingly, thrombin induced the recruitment and the association of Venus-Gαq and yPET-β-arrestin 2 with PAR1 independently on AT1R bringing more BRET acceptors in the vicinity of AT1R-Rluc along with those are directly interacting with AT1R-Rluc upon its activation by AngII (**Figure 7C**). This can be supported by our data with the selective blockade of receptors with their antagonists showing no full inhibition of the BRET signals induced by AngII/thrombin combined treatment. The remaining BRET signals may be due to thrombin activating PAR1 and promoting its interaction with Venus-Gαq and yPET-β-arrestin 2 which leads to BRET with AT1R-Rluc independently on AT1R-Rluc activation. This can be supported by the absence of any inhibition of thrombin-promoted BRET increase between AT1R-Rluc and yPET-β-arrestin by irbesartan (**Figure 6B**). Furthermore, our data also suggest the possibility of AT1R transactivation by thrombin and PAR1. According to this scenario, thrombin/PAR1 transactivate AT1R-Rluc resulting in a positive allosteric action on AngII-mediated receptor activation leading to increased AT1R-Rluc/Venus-Gαq and AT1R-Rluc/yPET-β-arrestin 2 interactions (Porrello et al., 2011; Mustafa et al., 2012). This was supported by two main observations: i) irbesartan blocking thrombin-promoted BRET increase between AT1R-Rluc/Venus-Gαq (**Figure 6A**) and ii) the fact that both irbesartan and SCH79797 fully blocked the potentiated BRET signals indicating that the active conformation of both PAR1 and AT1R was required for such BRET potentiation to occur upon AngII/thrombin combined treatment. Furthermore, our IP1 data strongly consolidated the transactivation of AT1R by thrombin and PAR1 with both antagonists fully inhibiting the potentiating IP1 response in HEK293 cells (**Figure 7C**) and irbesartan drastically inhibited the IP1 response elicited by the combined AngII/thrombin treatment in HT-29 cells (**Figure 7D**).

These different scenarios are based on the quantitative aspect of our BRET data and the stoichiometry of receptor/Gαq and yPET-β-arrestin 2 interactions assuming that BRET potentiation was related to the amount of Gαq and yPET-β-arrestin 2 being in the vicinity of and interacting with the receptors. Alternatively, our observations can be explained by the possibility of specific conformational changes induced by AngII/thrombin combined treatment on the AT1R/PAR1 complex. Accordingly, the co-activation of AT1R and PAR1 stabilizes both receptors in distinct conformations than those induced by the individual treatments and this confers a more favorable orientation and distance between AT1R-Rluc and Venus-Gαq and yPET-β-arrestin 2 leading to more efficient BRET responses to occur.

Surprisingly, the positive interaction between AngII/AT1R and thrombin/PAR1 concerning Gαq coupling, Gαq/inositol pathway, and β-arrestin recruitment contrasted well with the inhibitory effect of thrombin and PAR1 on AT1R internalization and its endosomal trafficking. Indeed, one would have also expected a potentiation of AT1R internalization by thrombin and PAR1 since GPCR-mediated β-arrestin recruitment and their internalization are two well-linked processes. Interestingly, such an inhibition of AT1R internalization and endosomal trafficking was observed only upon simultaneous co-activation of AT1R and PAR1 while the simple overexpression of PAR1 had no effect. This indicates the plasma membrane trapping of AT1R by thrombin-activated PAR1. Thus, the potentiation of Gαq coupling and β-arrestin recruitment observed in AT1R and PAR1 coexpressing cells and concomitantly treated with AngII and thrombin could be the consequence of the inhibition of AT1R internalization leading to more AT1R stacking at the plasma membrane. If this was true, the combined AngII/thrombin treatment may stabilize the receptors in a kind of locked active conformation which would not be followed by AT1R internalization and desensitization leading to stronger Gαq coupling and signaling and β-arrestin recruitment (**Figure 10C**). Such inhibition of AT1R internalization was previously reported *via* its heterodimerization with AngII type 2 receptor (AT2R) (Porrello et al., 2011). Another possible explanation is based on our data with the antagonists on BRET between AT1R-Rluc and yPET-β-arrestin 2 (**Figure 6B**). Indeed, SCH79797 but not irbesartan strongly inhibited thrombin-promoted BRET increase indicating that PAR1 but not AT1R activation was required for BRET between AT1R-Rluc and yPET-β-arrestin 2. This may indicate that yPET-β-arrestin 2 is recruited to PAR1 protomer only but not AT1R protomer within AT1R-PAR1 complex. If this is true, it explains the inhibitory effect of thrombin and PAR1 on β-arrestin-dependent AT1R internalization. It would be interesting to investigate more the impact of this on the intracellular AT1R-mediated signaling and its physiological relevance.

Despite our basic study did not directly address the different aspects in a more relevant physiological model, we believe that study paves the way for further investigation to confirm or not our data in more physiological models and contexts. Moreover, our data revealed for the first time the functional AT1R-PAR1 interaction that should be considered in the interpretation of the previous studies on the interaction between AngII and thrombin in the vascular system and the clinical approaches targeting RAS and thrombin. For instance, AT1R blockade has been shown to have beneficial effects in exercise-induced changes in coagulation and fibrinolysis in essential hypertension (Gavriilaki et al., 2014). Indeed, so far the therapeutic targeting of the vascular system and RAS is mostly based on AngII receptor blockers and ACE inhibitors but to a very less extent on thrombin and its receptor inhibitors (Walz et al., 1985; Heyndrickx, 1993; Serruys et al., 1995; Tsukuda et al., 2011; Urushihara et al., 2011). Moreover, in human clinical trials, both ACE and thrombin inhibitors have failed to decrease restenosis and this may be explained by a redundant signaling by other receptors such as the possibility of AngII/thrombin interaction at the level of their receptors as revealed by our study (Pratt Richard and Dzau Victor, 1996). Finally, the pivotal role of AngII/AT1R and thrombin/PAR1 in vasoconstriction and coagulation, respectively, and the tight link between these two processes in both physiological and pathophysiological situations further give credit to our *in vitro* investigation of the functional AT1R-PAR1 interaction with potential implication in the vascular system.

## Data Availability Statement

The raw data supporting the conclusions of this article will be made available by the authors, without undue reservation.

## Author Contributions 

IA performed research and analyzed data. AP, AA, and RI performed research. MAA conceived the project, analyzed the data, wrote the manuscript, and managed the project and its funding.

## Funding

This work was supported by the SURE Plus grant 2018-2019 (N°G00002745) and the Startup grant 2018-2020 (N°31S305) from the United Arab Emirates University.

## Conflict of Interest

The authors declare that the research was conducted in the absence of any commercial or financial relationships that could be construed as a potential conflict of interest.
